# Emerging Non-Canonical Functions and Regulation of p53

**DOI:** 10.3390/ijms19041015

**Published:** 2018-03-28

**Authors:** Atul Ranjan, Tomoo Iwakuma

**Affiliations:** 1 Department of Cancer Biology, The University of Kansas Cancer Center, University of Kansas Medical Center, Kansas City, KS 66010, USA; aranjan@kumc.edu; 2 Department of Hematology and Oncology, Children’s Mercy Hospital Research Institute, Kansas City, MO 64108, USA

The tumor suppressor p53 induces cell cycle arrest and/or apoptosis by transactivating numerous downstream target genes and also translocating to the mitochondrial outer membrane. These canonical p53 functions are considered as the major attribute to tumor suppression; however, recent findings suggest that other non-canonical p53 functions may be crucial for suppressing tumor progression. For example, a mutant p53 (p53^L25Q;W26S^) lacking the ability of inducing cell cycle arrest and apoptosis can still induce oncogene-induced senescence and inhibit Ras-induced tumor formation [1]. Also, knockin mice carrying a mutant p53 (p53^K117R;K161R;K162R^) that cannot induce cell cycle arrest, apoptosis, and senescence fail to develop tumors at an early age, unlike p53-null mice, likely because p53^K117R;K161R;K162R^ retains the abilities to regulate energy metabolism and reduce reactive oxygen species [2]. These results in mouse models strongly support the idea that non-canonical functions of p53 play significant roles in tumor suppression.

This special issue compiles emerging non-canonical functions and regulation of p53, including (1) novel mechanisms regulating the expression and activities of p53 and its family members, p63 and p73; (2) p53 proteoforms and binding potential of p53 to local DNA structures; (3) novel oncogenic pathways and stemness regulated by p53; (4) roles of p53 in non-canonical cell death; (5) roles of p53 in glucose, lipid, and nucleotide metabolism, and (6) roles of p53 in immunity. Understanding of these non-canonical p53 functions will help us with exact mechanisms by which p53 inhibits tumor progression and will accelerate the development of novel p53-tageted therapies against cancer and other diseases (Table 1). Many articles discuss about strategies to restore p53 activity in cancer cells with attenuated or no p53 activity and p53 mutations. However, such strategies may be affected by diverse and context-dependent functions of p53 and its family members.

## 1. Novel Mechanisms Regulating the Expression and Activities of p53 and Its Family Members, p63 and p73

Expression levels of p53 are regulated through multiple mechanisms. Particularly, importance of post-translational modifications (PTMs) of p53, such as phosphorylation, acetylation, and ubiquitination on p53 stability/degradation and activity, are well documented in the literature [3,4]. In this special issue, Herrero et al. [5] summarize molecular mechanisms of p53 deregulation in cancer, particularly focusing on multiple myeloma, including regulation of p53 expression levels by DNA methylation, micro RNAs (miRNAs), and alternative splicing, as well as alternative protein initiation.

Pollutri et al. [6] also discuss functional association between miRNAs and p53 in hepatocellular carcinoma (HCC). Many miRNAs have been shown to be transcriptionally regulated by p53 (p53-effectors), while there are some miRNAs that directly or indirectly regulate p53 expression levels (p53-regulators). Importantly, functional association between p53 and these miRNAs often forms positive or negative feedback loops (e.g., p53/miR-519d, -1228, -221) [7,8,9]. Moreover, in some cases, expression and functions of miRNAs are context-dependent. Nonetheless, the potential of microRNA-based therapeutic approaches should be considered for the treatment of HCC.

The levels of p53 are also regulated via protein translational machinery. Recent studies have identified internal ribosome entry site (IRES) sequences in the 5′-UTR of the *p53* mRNA to generate full-length p53 as well as downstream of the 1st AUG of the *p53* mRNA to generate ΔN-p53 [10]. Interestingly, there is a switch from cap-dependent translation to IRES-mediated translation of *p53* mRNA following DNA damage. Ji et al. [11] discuss about detailed process of the translational control of the p53 internal ribosome entry site (IRES) and targeting this machinery for cancer therapeutics.

Activity of p53 can be altered by its subcellular localization. For example, p53 is sequestered in the cytoplasm by p53-associated parkin-like cytoplasmic protein (PARC) [12] or Mortalin [13]. Carra et al. [14] mainly discuss their recent discovery of subcellular localization control of p53 by IκB-α, the *NFkBIA* gene product. In chronic myeloid leukemia (CML), a myeloproliferative disorder associated with the t(9:22) translocation producing BCR-ABL, IκB binds to the BCR-ABL, and this complex prevents p53 translocation into the nucleus [14,15]. Thus, disrupting IκB-α/p53 network could be used as a novel therapeutic strategy for CML.

Protein stability is one of the most critical factors that regulate p53 activity. Increasing evidence demonstrates that MDM2 is not the only E3 ubiquitin ligase to induce p53 degradation, while other ubiquitin ligases such as COP1, Pirh2, and CHIP can ubiquitinate and induce degradation of p53. Sane et al. [16] discuss about regulation of p53 by these ubiquitin ligases as well as functional regulation of these proteins, although it remains unclear which ligase is used for p53 ubiquitination in different cellular contexts. Nonetheless, these ligases and their regulatory networks can be considered as biomarkers and therapeutic targets for diverse types of cancers.

The tumor suppressor p53, as well as its family members p63 and p73, are derived from the *p63/p73* gene. Although all the members recognize the same target sequence in DNA, each has specialized function with some overlap. While p53 is expressed in most cells, p63 and p73 are specifically expressed in epithelial tissues of the ectoderm. All members have abilities to suppress tumor progression; however, p63 plays a role in epithelial development, metabolism, and senescence, while p73 regulates neurogenesis, pheromone signaling, and cerebrospinal fluid dynamics. Armstrong et al. [17] specially discuss about regulation of the tumor suppressor p63 and its oncogenic isoform ΔNp63 through multiple ubiquitin E3 ligases, including MDM2, Pirh2, Itch/AIP4, Nedd4, and WWP1. These E3 ligases can function as oncogenes or tumor suppressors depending on types of isoforms they target, which would likely be context-dependent [17].

## 2. p53 Proteoforms and Binding Potential of p53 to Local DNA Structures

Different forms of p53 structures can alter the ability of p53 to bind DNA, while different DNA structures also affect p53’s ability to interact with DNA. Uversky et al. [18] discuss about effects of multiple proteoforms of p53 by PTMs, isoforms, and mutations on the p53’s structures and functions. They propose p53 as an illustration of the protein structure–function continuum concept, in which generation of multiple proteoforms defines the ability of p53 to have a multitude of structurally and functionally different states. Additionally, Brazda et al. [19] summarize how different local DNA structures alter binding potential of wild-type and mutant p53. Indeed, interaction of p53 with local DNA structures (e.g., subtelomeric regions) contributes to prevention of DNA damage accumulation at human telomeres [20]. Also, p53 is shown to interact with genome locations that are not associated with transcriptional control [21]. Such diversity of p53-DNA binding may be involved in many cellular activities and non-canonical p53 functions. A deeper understanding of p53’s proteoforms, as well as its sequence-specific and structure-specific binding properties, will provide insights into the complexity of the p53 pathway and further help understand context-dependent functions of p53.

## 3. Novel Oncogenic Pathways and Stemness Regulated by p53

p53 is known to suppress tumor progression by activating other tumor suppressive pathways, whereas mutant p53 can promote tumorigenesis by inhibiting the tumor suppressive pathways. One of the pathways associated with p53 is the Hippo signaling pathway which prevents nuclear translocation of oncogenic molecules, YAP and TAZ. Ferraiuolo et al. [22] summarize direct and indirect association of wild-type p53 or mutant p53 with the Hippo signaling pathway. Intriguingly, YAP can act as a tumor suppressor in a context-dependent manner. Functional association of YAP with wild-type p53 results in tumor suppressive behaviors, whereas physical and functional interaction between YAP and mutant p53 promotes tumor progression.

Normal non-malignant stem cells play important role in the development and regeneration of organs, while cancer stem-like cells (CSCs) generate diverse population of cancer cells in a tumor with high malignant properties, including drug resistance, metastasis, and recurrence. Olivos et al. [23] discuss about the suppressive and promoting roles of wild-type p53 and gain-of-function (GOF) mutant p53, respectively, in stemness of non-malignant stem cells and CSCs, as well as in their microenvironment and CSC niche.

## 4. Roles of p53 in Non-Canonical Cell Death

An increasing number of studies have shown different forms of cell death machinery, many of which p53 is involved in. Apoptosis is the best-known form of cell death caused by p53, regulating tumor suppression, radiosensitivity, and chemosensitivity. The mechanism by which p53 induces caspase-dependent apoptotic cell death has been well described in other review articles [24,25,26]. Accumulating evidence demonstrates many alternative ways of cell death that is different from apoptotic cell death. These non-canonical cell death machineries can play crucial roles in inducing cell death as alternative modes when apoptosis fails. Ranjan et al. [27] summarize literatures showing the involvement of p53 in several non-canonical modes of cell death, including caspase-independent apoptosis (CIA), ferroptosis, necroptosis, autophagic cell death, mitotic catastrophe, paraptosis, pyroptosis, and efferocytosis. p53 induces these non-canonical forms of cell death through transcriptional regulation of its downstream targets and by direct binding with factors governing these cell death mechanisms. Thus, p53 induces various forms of cell deaths in different cellular contexts. Such diverse p53 functions to induce cell death greatly contribute to inhibiting tumor progression as the guardian of the genome. Hence, it would be important to explore therapeutic strategies to induce non-canonical cell death in cancer cells where apoptosis is frequently impaired.

## 5. Roles of p53 in Glucose, Lipid, and Nucleotide Metabolism

Metabolic reprogramming of cancer cells has been well appreciated (e.g., Warburg effect), allowing them to adapt in an environment, so that they can survive and proliferate under stressed conditions [28]. Recent advances in cancer research have suggested that metabolic reprogramming of cancer cells plays significant roles in malignant properties of cancer cells, including survival, proliferation, drug resistance, and metastasis. Importantly, these metabolic changes in cancer cells can be therapeutic targets. 

Itahana et al. [29] summarize the involvement of p53, mutant p53, and its family members p63 and p73 in cancer metabolism, mainly focusing on the metabolism of glucose which is the key source to provide energy. Generally, p53 inhibits glycolysis and enhances gluconeogenesis, mitochondrial oxidative phosphorylation, and the TCA cycle; however, in some cases, p53 is reported to function oppositely. Moreover, the involvement of p53 family members and their isoforms in glucose metabolism make more complex. All the members and isoforms of p53 family can act as a balance of maintaining glucose homeostasis in cells, thus likely being context-dependent. Such glucose metabolism regulation by p53 family members may also contribute to tumor suppression [29].

Parrales et al. [30] discuss about regulation of lipid metabolism by wild-type p53 and mutant p53, as well as their association with cancer progression. Overall, wild-type p53 enhances fatty acid oxidation and inhibits new lipid synthesis, whereas mutant p53 increases synthesis of fatty acid, cholesterol, and lipid droplets.

Schmidt et al. [31] discuss their recent findings that mutant p53 upregulates both nucleotide de novo synthesis and nucleoside salvage pathways as a novel GOF activity, promoting cancer progression [32]. This unexpected mutant p53 function is mainly mediated by its binding to an oncogenic transcription factor ETS2 in the promoters of multiple nucleotide metabolism genes (NMG). They also illustrate that cells harboring mutant p53 become dependent on deoxycytidine kinase (dCK), hence offering a new therapeutic opportunity for cancers carrying mutant p53.

## 6. Roles of p53 in Immunity

Accumulating evidence indicates that p53 and other tumor suppressors play important roles in immune response and inflammation either by directly or indirectly transactivating regulators involved in these processes [33,34]. Cui et al. [35] summarize literatures showing the involvement of p53 in inflammation, tumor microenvironment (TME), and antitumor immunity. Dysfunction of p53 not only enhances oncogenic properties of cancer cells, but also alters TME by skewing immune landscape (both innate and adaptive immunity) or properties of cancer-associated fibroblasts to favor cancer progression. They furthermore discuss about the effects of p53 reactivation from mutant p53 in the TME as an approach to promote antitumor immunity and overcome tumor-induced immune tolerance.

Fierabracci et al. [36] also describe the functional relationship of p53 with cancer and autoimmunity. Increasing number of studies show direct or indirect association of p53 with autoimmunity inhibition, using p53 knockout mouse models, as well as in human diseases, such as rheumatoid arthritis, systematic sclerosis, Sjὃgren syndrome, and Gravess disease. They likewise discuss the potential of p53 reactivation for suppressing both cancer progression and chronic inflammatory disorders leading to autoimmunity.

## 7. Summary

Accumulating evidence has demonstrated that p53 regulates numerous cellular activities by transcriptionally altering expression of downstream target genes or physically interacting with its binding partners, in addition to its well-characterized function in cell cycle progression and apoptosis. This special issue has collected multiple review articles that summarize the emerging non-canonical functions and regulation of p53, mutant p53, and its family members, from different viewpoints. Obviously, this issue could not cover all the non-canonical p53 functions or their regulatory mechanisms. Readers need to pay attention to studies that are not discussed in the current issue, as well as to further growing numbers of publications that show unexpected functions and regulation of p53 family members.

## Figures and Tables

**Table 1:** Summary of papers in the Special Issue “Emerging Non-Canonical Functions and Regulation of p53”.

Group	Title	Summary	Reference
(1) Novel mechanisms regulating the expression and activities of p53 and its family members, p63 and p73	Molecular Mechanisms of p53 Deregulation in Cancer: An Overview in Multiple Myeloma	In multiple myeloma, there are multiple ways to reduce p53 activity, including DNA methylation, miRNAs, and high levels of MDM2.	[5]
	*TP53*/MicroRNA Interplay in Hepatocellular Carcinoma	Expression levels of p53 are directly or indirectly altered by several miRNAs, while p53 regulates expression of multiple effector miRNAs.	[6]
	Targeting IRES-Mediated p53 Synthesis for Cancer Diagnosis and Therapeutics	There are IRES sequences in the 5′-UTR of the *p53* mRNA and downstream of the 1st AUG of the *p53* mRNA which regulate p53 translation and its protein levels in cells.	[11]
	Mechanisms of p53 Functional De-Regulation: Role of the IκB-α/p53 Complex	The BCR-ABL/IκB complex prevents p53 translocation into the nucleus in CML.	[14]
	Essential Roles of E3 Ubiquitin Ligases in p53 Regulation	Levels of p53 are regulated by multiple ubiquitin ligases which could be used as biomarkers or targeted for cancer therapy.	[16]
	The Regulation of Tumor Suppressor p63 by the Ubiquitin-Proteasome System	Several ubiquitin ligases target p63 and/or ∆p63 for degradation, of which HECT-containing E3 ligases (Itch/AIP4, Nedd4, and WWP1) do not induce degradation of p53.	[17]
(2) p53 proteoforms and binding potential of p53 to local DNA structures	p53 Proteoforms and Intrinsic Disorder: An Illustration of the Protein Structure–Function Continuum Concept	PTMs, alternative splicing, alternative promoter usage, alternative initiation of protein translation, and mutations of p53 can generate diverse structures and functions of p53.	[18]
	Recognition of Local DNA Structures by p53 Protein	Wild-type p53 binds to consensus target sequences in linear B-DNA as well as in cruciform DNA structure. Moreover, wild-type p53 and mutant p53 could bind to other local DNA structures including quadruplexes, triplexes, DNA loops, bulged DNA, and hemicatenane DNA by acting as DNA topology-modulating factors.	[19]
(3) Novel oncogenic pathways and stemness regulated by p53	Mutant p53 Protein and the Hippo Transducers YAP and TAZ: A Critical Oncogenic Node in Human Cancers	The Hippo pathway prevents nuclear translocation of oncogenic proteins, YAP and TAZ. Generally, association of YAP with wild-type p53 suppresses tumorigenesis, whereas its interaction with mutant p53 promotes tumor progression.	[22]
	Emerging Non-Canonical Functions and Regulation by p53: p53 and Stemness	Wild-type p53, its isoforms, mutant p53, and their regulatory networks play roles in stemness of normal and CSCs. Restoring p53 function in CSCs could be a novel therapeutic strategy for cancer.	[23]
(4) Roles of p53 in non-canonical cell death	Non-Canonical Cell Death Induced by p53	p53 is involved in not only caspase-dependent cell death, but also non-canonical cell death including CIA, ferroptosis, necroptosis, autophagic cell death, mitotic catastrophe, paraptosis, pyroptosis, and efferocytosis.	[27]
(5) Roles of p53 in glucose, lipid, and nucleotide metabolism	Emerging Roles of p53 family members in Glucose Metabolism	Wild-type p53, mutant p53, and its family members play vital roles in glucose metabolism, which possibly contributes to tumor suppression or progression.	[29]
	p53 as a Regulator of Lipid Metabolism in Cancer	Wild-type p53 inhibits the fatty acid synthesis and lipid accumulation, whereas mutant p53 enhances these processes. Also, mutant p53 upregulates mevalonate pathway enzymes.	[30]
	Control of Nucleotide Metabolism Enables Mutant p53’s Oncogenic Gain-of-Function Activity	Mutant p53 transactivates multiple nucleotide metabolism genes involved in both the nucleotide de novo synthesis and salvage pathways by associating with ETS2 in their promoter regions.	[31]
(6) Roles of p53 in immunity	Immunomodulatory Function of the Tumor Suppressor p53 in Host Immune Response and the Tumor Microenvironment	Status of p53 in cancer cells, as well as that in tumor microenvironment, can affect host immune response.	[35]
	The Double Role of p53 in Cancer and Autoimmunity and Its Potential as Therapeutic Target	p53 plays a role in inflammation and autoimmune conditions. Reactivating p53 could not only suppress tumor progression, but also induce anti-tumor inflammatory response.	[36]
